# Metrics of care and cardiovascular outcomes in patients with ST-elevation myocardial infarction treated with pharmacoinvasive strategy: a decade-long network in a populous city in Brazil

**DOI:** 10.1186/s12872-023-03340-6

**Published:** 2023-06-15

**Authors:** Pedro Ivo De Marqui Moraes, Attilio Galhardo, Adriano Henrique Pereira Barbosa, Jose Marconi Almeida de Sousa, Claudia Maria Rodrigues Alves, Henrique Tria Bianco, Rui Manuel dos Santos Povoa, Edson Stefanini, Iran Goncalves, Dirceu Rodrigues de Almeida, Francisco Antonio Helfenstein Fonseca, Maria Cristina de Oliveira Izar, Valdir Ambrosio Moises, Renato Delascio Lopes, Antonio Carlos Carvalho, Adriano Caixeta

**Affiliations:** 1 Discipline of Cardiology, Department of Medicine, Universidade Federal de Sao Paulo, Rua Napoleao de Barros, 715, Ground Floor, Hospital Sao Paulo, Vila Clementino, Sao Paulo, SP CEP (ZIP) 04024-002 Brazil; 2 Duke University Hospital, Duke Clinical Research Institute, DUMC, 2400 Pratt Street, Terrace Level Room 0311, Box 3850, Durham, NC 27705 USA; 3 Hospital Israelita Albert Einstein, Av. Albert Einstein, 627/701 - Morumbi, Sao Paulo, SP CEP (ZIP) 05652-900 Brazil

**Keywords:** ST Elevation Myocardial Infarction, Thrombolytic Therapy, Myocardial Reperfusion, Pharmacoinvasive strategy

## Abstract

**Background:**

Pharmacoinvasive strategy is an effective myocardial reperfusion therapy when primary percutaneous coronary intervention (p-PCI) cannot be performed in a timely manner.

**Methods:**

Authors sought to evaluate metrics of care and cardiovascular outcomes in a decade-long registry of a pharmacoinvasive strategy network for the treatment of ST-elevation myocardial infarction (STEMI). Data from a local network including patients undergoing fibrinolysis in county hospitals and systematically transferred to the tertiary center were accessed from March 2010 to September 2020. Numerical variables were described as median and interquartile range. Area under the curve (AUC-ROC) was used to analyze the predictive value of TIMI and GRACE scores for in-hospital mortality.

**Results:**

A total of 2,710 consecutive STEMI patients aged 59 [51–66] years, 815 women (30.1%) and 837 individuals with diabetes (30.9%) were analyzed. The time from symptom onset to first-medical-contact was 120 [60–210] minutes and the door-to-needle time was 70 [43–115] minutes. Rescue-PCI was required in 929 patients (34.3%), in whom the fibrinolytic-catheterization time was 7.2 [4.9–11.8] hours, compared to 15.7 [6.8–22,7] hours in those who had successful lytic reperfusion. All cause in-hospital mortality occurred in 151 (5.6%) patients, reinfarction in 47 (1.7%) and ischemic stroke in 33 (1.2%). Major bleeding occurred in 73 (2.7%) patients, including 19 (0.7%) cases of intracranial bleeding. C-statistic confirmed that both scores had high predictive values for in-hospital mortality, demonstrated by TIMI AUC-ROC of 0.80 [0,77–0.84] and GRACE AUC-ROC of 0.86 [0.83—0.89].

**Conclusion:**

In a real world registry of a decade-long network for the treatment of ST-elevation myocardial infarction based on the pharmacoinvasive strategy, low rates of in-hospital mortality and cardiovascular outcomes were observed, despite prolonged time metrics for both fibrinolytic therapy and rescue-PCI. Register Clinicaltrials.gov NCT02090712 date of first registration 18/03/2014.

## Background

Pharmacoinvasive strategy (PhIS) is especially valuable in overcoming barriers that withhold performing primary percutaneous coronary intervention (p-PCI) within the recommended 90 to 120 min from arrival to the first-medical-contact, circumstances that include distance and geographic blockades to the cardiac catheterization center, unavailability of ambulances, excessive traffic, poorly adapted logistics and lack of team training [1–5].

Systematic and early (2 to 24 h) angiography is recommended after successful fibrinolysis, mainly stated by a reduction greater than 50% of ST-elevation and symptom relief [6–8]. In cases of failed fibrinolysis, or if there is evidence of reocclusion with recurrence of ST-elevation, immediate angiography and rescue-PCI is indicated since re-administration of fibrinolytic has not been shown beneficial [9, 10].

Randomized trials and meta-analyses have proved that PhIS reduces rates of reinfarction and recurrent ischemia compared to the watchful waiting strategy, in which cardiac catheterization is performed in patients with recurrent ischemia, electrical or hemodynamic instability after fibrinolysis [11–15].

In Brazil, data from the Unified Health System (SUS) indicate that 35% of deaths are caused by cardiovascular diseases and in-hospital mortality from ST-elevation myocardial infarction (STEMI) in public hospitals ranged from 10.7% to 13.4% between 2010 and 2016 [16], although many institutions report better results, especially considering the hiatus between public and private healthcare systems [17–20].

In contrast to the vast advances in STEMI therapy in recent decades, knowledge gaps remain regarding the practical results of implementing regional networks to enhance early reperfusion, particularly in low or middle-income countries, where PhIS is attractive given the potential obstacles for primary PCI [21–23].

## Methods

### Aims

The authors sought to analyze the metrics of care and subsequent cardiovascular outcomes in patients with ST-elevation myocardial infarction treated in a local network based on the pharmacoinvasive strategy during a decade-long registry.

The secondary objectives were to validate the predictive values of the classical prognostic scores Thrombolysis in Myocardial Infarction (TIMI) and Global Registry of Acute Coronary Events (GRACE) and to assess whether the time interval for myocardial reperfusion has improved over the years.

### Patients and the STEMI network

Patients with a presumed diagnosis of STEMI initially treated in one of the 14 county hospitals that composed the STEMI network in the city of Sao Paulo, Brazil, were systematically transferred to the tertiary center. The hub-and-spoke urban distance ranged from 7 to 32 km, with an average distance of 16.2 km. Due to an expected operational delay of more than 120 min for p-PCI, in non-compliance with current guidelines, the pharmacoinvasive strategy was the default reperfusion therapy. The present study constitutes a prospective registry of all-comers patients over 18 years-old with an initially presumed diagnosis of STEMI treated in the local network [24].

Intravenous full doses of the fibrinolytics alteplase and tenecteplase were based on patient weight and half-dose was given to patients over 75 years of age. Initial standardized adjuvant treatment consisted of aspirin 300 mg, clopidogrel 300 mg and enoxaparin 30 mg intravenously followed by 1 mg/kg subcutaneously. With exception of patients over 75 years-old, who were prescribed clopidogrel 75 mg and enoxaparin adjusted to 0.75 mg/kg subcutaneously (without the 30 mg intravenous loading dose). After fibrinolysis, patients were systematically transferred to the tertiary hospital aiming cardiac catheterization between 2 and 24 h, or as soon as possible in the absence of reperfusion criteria [6, 10].

Nitrates, opioid derivatives, betablockers, renin–angiotensin–aldosterone system inhibitors and statins were prescribed according to the attending cardiologist discretion following current recommendations, without specific standardization. Laboratory tests included blood count, sodium, potassium, urea, creatinine, troponin (high sensitivity Troponin T Roche® 99th percentile: 14 pg/mL), transaminases, lipid profile and blood glucose. Complications related to STEMI and cardiac catheterization were recorded during the hospital stay.

The data was collected prospectively during the tertiary hospital internment for the index event by trained Cardiology resident physicians with digital storage on the Research Electronic Data Capture – Redcap platform [25]. The review of the angiographic characteristics of all patients was performed by a single experienced Interventional Cardiologist at the tertiary center.

### Definitions

The diagnosis of STEMI was based on clinical features of myocardial ischemia associated with ST-segment elevation ≥ 1 mm in at least 2 contiguous leads, or ≥ 2 mm in leads V2 and V3, or a new or presumably new left bundle branch block. Chronic kidney failure was defined by creatinine clearance < 60 ml/min estimated by the Cockroft-Gault equation.

Reperfusion criteria were evaluated 60 to 90 min after the initiation of intravenous fibrinolytic as reduction of more than 50% in the amplitude of the ST-segment elevation accompanied by symptom relief. In the absence of reperfusion criteria, patients were referred for rescue-PCI. Door-to-needle time was defined as the interval between arrival at the emergency department and lytic therapy. The predictive scores Thrombolysis in Myocardial Infarction for STEMI (TIMI), Global Registry of Acute Coronary Events (GRACE) and CRUSADE bleeding score were calculated at the time of county hospital admission.

Post-STEMI acute heart failure was clinically defined by the Framingham criteria and cardiogenic shock was assessed by clinical and laboratory criteria that included systolic blood pressure less than or equal to 90 mmHg sustained for more than thirty minutes, or need for vasoactive drugs, associated with changes in at least one tissue perfusion parameter (prolonged capillary refill time, increased arterial lactate, metabolic acidosis, decreased central venous saturation, oliguria, sensory impairment or reduced cardiac index) [26]. Major bleeding was defined as life-threatening by the Bleeding Academic Research Consortium (BARC classification) 3 to 5.

### Statistical analysis

Categorical variables were described as absolute and relative frequencies and compared using the chi-square test. Continuous variables were evaluated for normality through asymmetry (skewness) and kurtosis (kurtosis) values. Variables with normal distribution were described as mean and standard deviation (SD) and compared using the Student's t test. Non-normal variables were described as median and interquartile range [IQ] and compared by the Mann–Whitney test.

The area under the curve (AUC) obtained from the Receiver Operating Characteristic (ROC) curves were developed to analyze the predictive value (c-statistics) of the TIMI and GRACE scores for in-hospital mortality, compared by the DeLong test when appropriate.

In order to assess whether there was a significant modification in the time interval for myocardial reperfusion over the decade, the total period was divided into tertiles of 42 months each, and the door-to-needle and rescue-PCI times were then compared between tertiles by the Wilcoxon test.

All tests were two-tailed and *p*-values < 0.05 were considered significant. Statistical analyzes were performed using SPSS software (IBM Corp. SPSS Statistics for Windows, version 24.0. Armonk, NY).

## Results

### Demographic and clinical characteristics

**Fig. 1:**
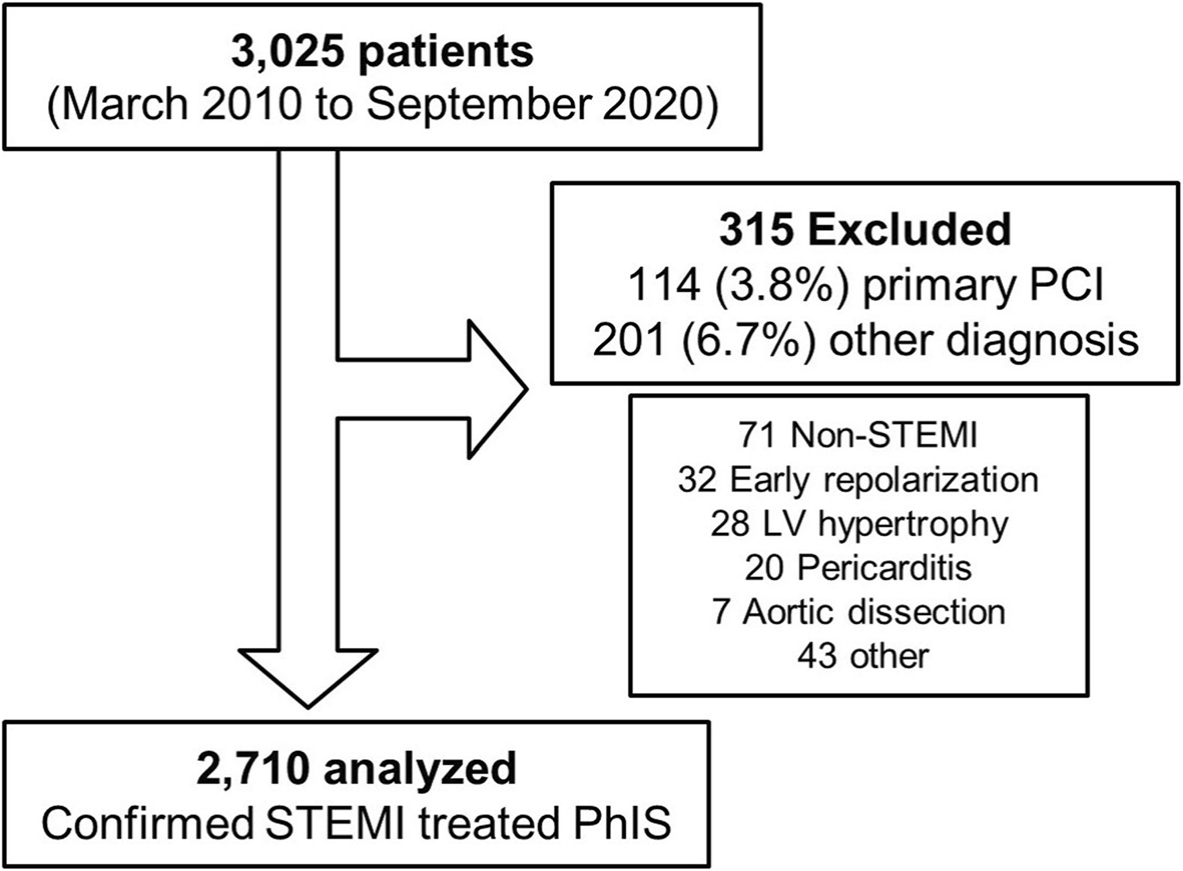
Flowchart of patients treated in the local STEMI network. PCI: percutaneous coronary intervention; LV: left ventricle; PhIS: pharmacoinvasive strategy

A total of 3,025 patients were treated in the local STEMI network from March 2010 to September 2020. We excluded from the analyses 201 (6.7%) patients with non-STEMI and 114 (3.8%) transferred for p-PCI, therefore analyzing 2,710 consecutive STEMI patients treated according to pharmacoinvasive strategy (Fig. 1). There was no significant difference between the analyzed and excluded groups in terms of in-hospital mortality (5.6% vs. 6.4% respectively, p = 0.73).

Median age was 59 [51–66] years-old and 815 (30.1%) were women. Initial presentation was typical angina in 2506 (92.5%), anginal equivalent in 168 (6.2%) and cardiorespiratory arrest in 36 (1.3%) cases. Hypertension was present in 1,632 (60.2%), dyslipidemia in 1,335 (49.3%), diabetes mellitus in 837 (30.9%), previous myocardial infarction in 261 (9.6%) and chronic kidney failure in 183 (6.8%) (Table 1).

**Table 1:** Baseline characteristics of STEMI patients treated in the local network

**Demography**	
Age, years	59 [51–66]
Female, n (%)	815 (30.1%)
BMI, Kg/m^2^	26.3 [23.9–29.4]
**Killip pre-fibrinolysis**	
I	2,539 (94.0%)
II-III	145 (5.4%)
IV	16 (0.6%)
**Comorbidities**	
Hypertension	1,632 (60.2%)
Dyslipidemia	1,335 (49.3%)
Diabetes mellitus	837 (30.9%)
Family history of AMI	566 (20.9%)
Obesity	526 (19.4%)
Current smoking	1,002 (36.9%)
Previous AMI	261 (9.6%)
Chronic kidney failure	183 (6.8%)
PAD	117 (4.3%)
Previous stroke	109 (4.0%)
Previous CABG	47 (1.7%)
**Scores**	**Mean ± SD**
TIMI	3.7 ± 2.3
GRACE	116.6 ± 38.4
CRUSADE	26.9 ± 14.7
Estimated bleeding risk	5.5% (± 2.9%)

*BMI* body mass index, *AMI* acute myocardial infarction, *PAD* peripheral artery disease, *CABG* coronary artery bypass graft, *RV* right ventricle, *SD* standard deviation

According to the Killip-Kimball classification, most patients had no signs of congestive heart failure prior fibrinolysis, with 2,539 (94.0%) patients in class I, 145 (5.4%) in class II or III and 16 (0.6%) in class IV cardiogenic shock. Initial ECG exhibit anterior wall involvement in 1,268 (46.8%) and fibrinolytic therapy was performed with tenecteplase in 2629 (97.0%), alteplase in 57 (2.1%) and streptokinase in 23 (0.9%) patients.

Admission prognostic assessment revealed a mean TIMI score of 3.7 (± 2.3), GRACE score of 116.6 (± 38.4) and CRUSADE score of 26.9 (± 14.7), which predicted a 5.5% (± 2.9%) major bleeding risk.

Laboratory tests collected on admission to the tertiary center showed median values of troponin 3,900 [1,534—9,200] pg/mL, glucose 123 [103—164] mg/dL, total cholesterol 196 [166—228] mg/dL, HDL-cholesterol 40 [33—48] mg/dL, LDL-cholesterol 124 [100—152] mg/dL and triglycerides 131 [ 92—190] mg/dL.

### Metrics of care

The time interval between symptom onset to the first-medical-contact was 120 [60—210] minutes and the door-to-needle time was 70 [43—115] minutes. Rescue-PCI was required in 929 (34.3%) patients, in whom the median fibrinolytic-catheterization time was 7.2 [4.9—11.8] hours compared to 15.7 [6.8—22.7] hours in those who had successful reperfusion criteria (Table 2).

**Table 2:** Temporal metrics of STEMI patients treated in the local network

Time interval	Median [IQ]
Symptoms to first-medical-contact	120 min [60–210 min]
Door-to-needle	70 min [43–115 min]
Fibrinolysis-angiography in PhIS group	15.7 h [6.8–22.7 h]
Fibrinolysis-angiography in rescue-PCI group	7.2 h [4.9–11.8 h]

*IQ* interquartile range, *PhIS* pharmacoinvasive strategy, *PCI* percutaneous coronary intervention

Over the decade-registry, there was no significant change in the door-to-needle time when comparing the 42-month long tertiles, respectively 65 [42–100] min, 71 [45–110] min and 73 [45–120] min; *p*-value = 0,15. Time for rescue-PCI also showed no significant difference between tertiles, respectively 6.9 [4.6–11.2] hours, 7.1 [4.8–11.8] hours and 7.2 [4.9–12.0] hours; *p*-value = 0,28.

The infarct-related artery (IRA) was the left anterior descending in 1204 (44.4%) patients, right coronary in 1065 (39.3%), circumflex-marginal in 256 (9.5%), left main disease in 9 (0.3%) and could not be determined in 130 (4.8%). The remaining 46 (1.7%) patients did not undergo cardiac catheterization, either due to early death or to a contraindication to the procedure.

Coronary TIMI flow graded 3 through the IRA was observed in 1,614 (59.5%) patients in the initial angiography and in 2,106 (77.7%) at the end of the procedure, after PCI, if needed. Bare-metal stents were implanted in 1,843 (91.0%) patients and drug-eluting stents in 182 (9.0%).

Following fibrinolysis, 2025 (74.7%) patients were treated with percutaneous coronary intervention, 594 (21.9%) received optimal medical therapy without need for PCI and 91 (3.4%) underwent coronary artery bypass graft (CABG) surgery (Table 3).

**Table 3:** Angiographic characteristics of STEMI patients treated in the local network

**IRA**	
Left anterior descending	1,204 (44.4%)
Right coronary	1,065 (39.3%)
Circumflex or marginal artery	256 (9.5%)
Left main	9 (0.3%)
Undetermined	130 (4.8%)
Angiography not performed	46 (1.7%)
**Initial IRA coronary TIMI flow**	
TIMI 0	528 (19.5%)
TIMI 1	126 (4.6%)
TIMI 2	410 (15.1%)
TIMI 3	1,614 (59.6%)
**Final IRA coronary TIMI flow**	
TIMI 0	153 (5.6%)
TIMI 1	40 (1.5%)
TIMI 2	379 (14.0%)
TIMI 3	2.106 (77.7%)
**Revascularization**	
Fibrinolysis followed by PCI	2,025 (74.7%)
Fibrinolysis without need for PCI	594 (21.9%)
Fibrinolysis followed by CABG	91 (3.4%)
**Other features**	
Multivessel disease	602 (22.6%)
Bare metal stenting	1,843 (91.0%)

*IRA* infarct-related artery, *TIMI* Thrombolysis In Myocardial Infarction, *PCI* percutaneous coronary intervention, *CABG* coronary artery bypass graft

### Cardiovascular outcomes

All cause in-hospital mortality occurred in 151 (5.6%) patients, reinfarction in 47 (1.7%) and ischemic stroke in 33 (1.2%). Major bleeding occurred in 73 (2.7%) patients, including 19 (0.7%) cases of intracranial bleeding. Minor bleeding, mostly related to hematoma at the puncture site of cardiac catheterization, occurred in 97 (3.6%) patients.

The most frequent complication related to STEMI was acute heart failure, found in 1,162 (42.9%) patients. Cardiogenic shock occurred in 241 (8.9%) cases and isolated right ventricle heart failure in 124 (4.6%). Complex ventricular arrhythmias followed by sudden cardiac death, aborted or not, occurred in 228 (8.4%) cases, complete atrioventricular block in 139 (5.1%) and de novo atrial fibrillation in 94 (3.5%) (Table 4).

**Table 4:** In-hospital cardiovascular outcomes of STEMI patients treated in the local network

**Ischemic outcomes**	N (%)
All-cause mortality	151 (5.6%)
Reinfarction	47 (1.7%)
Stroke	33 (1.2%)
**Heart failure**	
Acute heart failure	1,162 (42.9%)
Cardiogenic shock	241 (8.9%)
Isolated RV heart failure	124 (4.6%)
**Hemorrhagic outcomes**	N (%)
Major bleeding	97 (3.6%)
Minor bleeding	73 (2.7%)
Intra-cranial bleeding	19 (0.7%)
**Arrhythmias**	
Ventricular arrhythmias with SCD (aborted or not)	228 (8.4%)
Complete AV block	139 (5.1%)
De novo atrial fibrillation	94 (3.5%)

*RV* right ventricle, *SCD* sudden cardiac death, *AV* atrioventricular

**Fig. 2:**
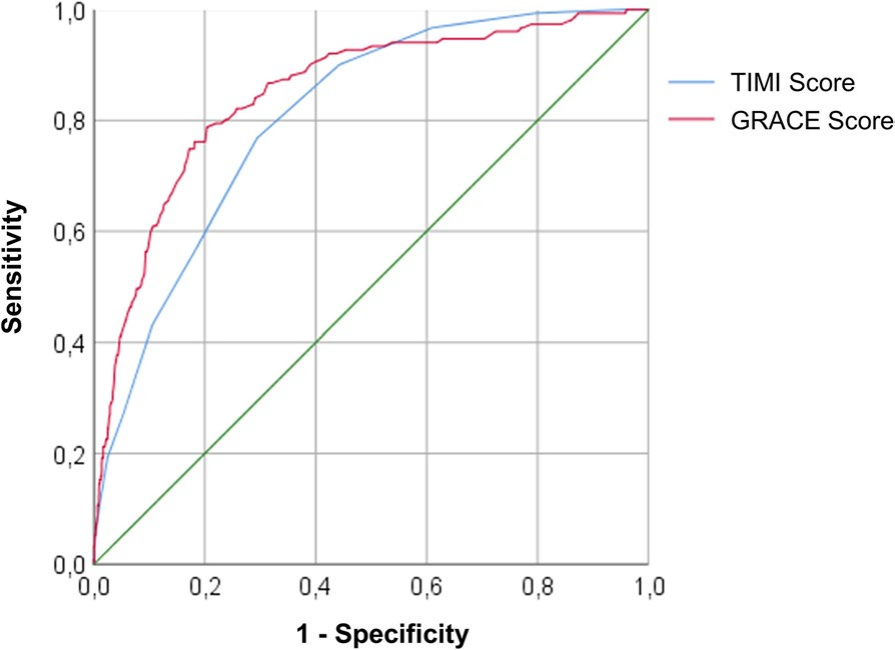
ROC curves of TIMI and GRACE scores for in-hospital mortality prediction. AUC-ROC TIMI score 0.804 ± 0.016 (95% CI 0.772—0.836), p < 0.001. AUC-ROC GRACE score 0.859 ± 0.016 (95% CI 0.828—0.890), p < 0.001. AUC-ROC TIMI x GRACE DeLong test *p*-value 0,78

Patients admitted to the STEMI network could be considered at moderate baseline risk for major ischemic events during hospitalization according to prognostic scores of TIMI (3.7 ± 2.3) and GRACE (116.6 ± 38.4). C-statistic confirmed that both scores had high predictive values for in-hospital mortality, demonstrated by TIMI AUC-ROC of 0.80 (95% confidence interval 0,77—0.84 *p*-value < 0.01) and GRACE AUC-ROC of 0.86 (95% confidence interval 0.83—0.89 *p*-value < 0.01) (Fig. 2), with no statistical difference between them (DeLong test *p*-value 0,78).

**Fig. 3:**
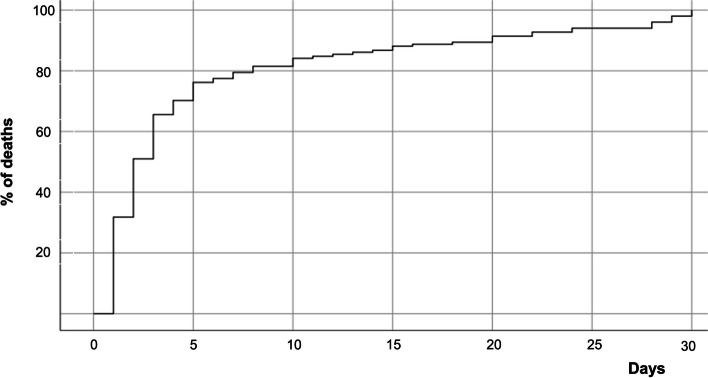
Time elapsed until in-hospital death

The time elapsed until in-hospital mortality warns to the precocity of STEMI complications, as more than half of the deaths occurred in the first two days of hospitalization and 75% of them in the first five days (Fig. 3).

Mechanical complications, diagnosed by echocardiography or necropsy, were identified in 13 cases (0.5%) of severe mitral regurgitation, 4 cases (0.14%) of ventricular septal defect and 2 cases (0.07%) of left ventricular free wall rupture. Necropsy was performed in only 14 cases of death.

## Discussion

This registry of STEMI patients treated with a pharmacoinvasive strategy includes a population with a high prevalence of cardiovascular risk factors and significantly younger when compared to other similar registries [27].

The median time of seventy minutes between the first-medical-contact and the initiation of fibrinolytic therapy largely exceeded the recommended by guidelines, with less than 15% of patients reaching the goal of a door-to-needle time up to thirty minutes. This delay has prognostic implications and is even more alarming considering the relatively short time, median two hours, in which patients arrived at the county hospital from the symptom onset [5, 23].

Throughout the years of the local STEMI network, time metrics for lytic therapy and rescue-PCI did not improve. Despite periodic training of health professionals, the authors considered preponderant factors for the sustained delay in myocardial reperfusion therapy, such as the scarcity of resources in the public health system, unavailability of ambulances for prompt removal and high turnover of physicians in county hospitals. Efforts to structure logistics, rapid transfer, mobilization and training of healthcare teams combine an imminent need to improve metrics of care [28, 29]. Mortality rates also remained steady over the observation period, possibly due to the constancy of metrics of care and the standardization of pharmacological and interventional therapy utilized in the STEMI network over the decade.

During initial cardiac catheterization, coronary TIMI-flow 2 or 3 through the infarct-related artery was observed in 2024 (74.7%) patients, in agreement with previous publications showing a chance greater than 70% for restoration of adequate coronary flow after fibrinolysis with t-PA and tenecteplase [30, 31]. After percutaneous coronary intervention, when indicated, TIMI 2 or 3 coronary flow was achieved in 2,485 (91.7%) cases, highlighting the valuable combination of the two reperfusion therapies for myocardial salvage.

In international registries, 30-day mortality in STEMI treatment networks ranges from 4.2% to 13% [27]. In Brazil, acute coronary syndromes are the third leading cause of hospitalization and mortality rates above 10% have been reported in some regions [16, 18]. Despite the limited resources, logistics and care metrics, relatively low rates of in-hospital mortality were observed in patients treated by the local STEMI network.

The bleeding risk associated with fibrinolytics imposes a historical barrier to reperfusion treatment in STEMI. The estimated risk, predicted by the CRUSADE score, indicated a low risk profile for bleeding, on average of 5.5% (± 2.9%). This matter was confirmed in practice, with 73 (2.7%) patients presenting major bleeding, including 19 (0.7%) cases of intra-cranial bleeding, numbers comparable to historical series that showed rates of hemorrhagic stroke after fibrinolysis ranging between 0.3 and 1.3% [30, 31].

The high prevalence of acute heart failure after STEMI (42.9%), which could be explained by the delay in reperfusion therapy, may also incorporate the transient phenomenon of stunned myocardium and does not necessarily represent the number of patients who will maintain chronic heart failure after hospital discharge. This finding reinforces the relevance of rapid reperfusion and for the surveillance of acute heart failure after STEMI, so that appropriate therapeutic measures can be instituted.

Cardiogenic shock is the most common cause of in-hospital death in patients with acute myocardial infarction. Even with contemporary therapeutic advances and adequate reperfusion rates, cardiogenic shock after STEMI has an 8 to 13% incidence and mortality rates of up to 50% are reported in international registries [32, 33]. Data from the pharmacoinvasive network in the city of Sao Paulo show very similar results.

Importantly, the analysis also allows the validation of the classical TIMI and GRACE scores for predicting in-hospital mortality in the pharmacoinvasive strategy scenario.

The COVID-19 pandemic outbroke in Brazil in March 2020 and imposed major challenges and constraints on global healthcare. Over the pandemic months from March to September 2020, only 65 STEMI patients were seen, with a mortality rate of 3.1% (2 patients). The authors decided to include these cases to document the last fully operating six months of the local STEMI network.

### Limitations

The observational nature of this registry provides relevant descriptive data and real-world metrics, although it might not constitute the ideal design for clinical decision-making and to inform guidelines. The presence of a single tertiary center may also contribute to a lower external validation of the results.

The low rate of mechanical complications after STEMI, less than 1%, is probably underestimated due to the low number of necropsies performed in patients who evolved to sudden non-aborted cardiac death.

The absence of descriptive data on medications, such as beta-blockers, renin–angiotensin–aldosterone system inhibitors, statins and antidiabetics constitutes an important limitation of this study. Optimal medical therapy is a metric of quality of care and directly influences cardiovascular outcomes, but it could not be assessed due to a record gap.

### Perspectives

The cornerstone for STEMI treatment is early and effective reperfusion aiming myocardial salvage. The use of fibrinolytics from the second half of the twentieth century onwards, and the advent and modernization of percutaneous coronary intervention techniques contributed to a significant reduction in mortality and cardiovascular complications [34].

However, in many locations, due to operational, geographic, financial, and technical obstacles, failures in reperfusion strategies lead to unacceptably high levels of death from STEMI. The establishment, expansion and improvement of STEMI treatment networks based on a pharmacoinvasive strategy accumulate a large body of evidence and constitute a feasible and effective means to reduce mortality and cardiovascular complications, especially when p-PCI cannot be performed in a timely manner [4, 8, 17, 22, 35–37].

## Conclusion

In the decade-long registry of a local network for the treatment of STEMI based on the pharmacoinvasive strategy, low rates of in-hospital mortality and of both ischemic and hemorrhagic cardiovascular outcomes were observed, despite prolonged time metrics for fibrinolytic therapy and rescue-PCI.

## Data Availability

Further data information and materials will be shared on reasonable request to the corresponding author.
